# Genetic Modifiers and Rare Mendelian Disease

**DOI:** 10.3390/genes11030239

**Published:** 2020-02-25

**Authors:** K. M. Tahsin Hassan Rahit, Maja Tarailo-Graovac

**Affiliations:** 1 Departments of Biochemistry, Molecular Biology and Medical Genetics, Cumming School of Medicine, University of Calgary, Calgary, AB T2N 4N1, Canada; kmtahsinhassan.rahit@ucalgary.ca; 2 Alberta Children’s Hospital Research Institute, University of Calgary, Calgary, AB T2N 4N1, Canada

**Keywords:** genetic modifier, mendelian disease, rare disease, GWAS, genome sequencing, genetic interaction, penetrance, expressivity, phenotypic variability, bioinformatics

## Abstract

Despite advances in high-throughput sequencing that have revolutionized the discovery of gene defects in rare Mendelian diseases, there are still gaps in translating individual genome variation to observed phenotypic outcomes. While we continue to improve genomics approaches to identify primary disease-causing variants, it is evident that no genetic variant acts alone. In other words, some other variants in the genome (genetic modifiers) may alleviate (suppress) or exacerbate (enhance) the severity of the disease, resulting in the variability of phenotypic outcomes. Thus, to truly understand the disease, we need to consider how the disease-causing variants interact with the rest of the genome in an individual. Here, we review the current state-of-the-field in the identification of genetic modifiers in rare Mendelian diseases and discuss the potential for future approaches that could bridge the existing gap.

## 1. Introduction

Diseases that follow Mendelian patterns of inheritance are known as Mendelian disorders. Approximately 80% of all rare diseases are genetic in origin, and most of these diseases are monogenic/Mendelian [1,2]. Rare diseases are individually rare, but according to the estimate, there are 400 million people all over the world suffering from around 7000 different rare diseases [1,3,4,5]. Despite being monogenic, the genetic basis of more than half of all identified Mendelian diseases remains elusive [2,4,6]. Even in cases where the causal disease gene is known, these diseases can display variable phenotypes, even in patients with the same causal mutation (e.g., siblings [7,8,9,10]), which poses diagnostic and patient management challenges [11]. Conversely, researchers have analyzed multiple human genome/exome data and found evidence of disease-causing genotypes in individuals with no reported disease presentations [12,13]. These large-scale genome-wide sequencing studies provide further information on rare disease penetrance and expressivity, as well as a potential effect of genetic modifiers.

Identifying the genetic origin of a specific phenotype is not so straightforward, as the genotypes and phenotypes may not have a linear relationship. This is partially due to genetic interactions that play a crucial role in controlling the functional dependency between genes [14] and can result in phenotypic variability. Numerous things can affect the genetic pathway to produce unexpected phenotypes. Genetic modifiers, defined as genetic variants that can modify the phenotypic outcome of the primary disease-causing variant, are one such example. They can increase (known as an enhancer) or decrease (known as a suppressor) the severity of the disease condition but may not be disease-causing themselves. Modifier variants can change a target gene’s phenotype by having a genetic, biochemical, or functional interaction with one or more target gene(s), or gene product(s) [15]. The degree of the effect of the modifiers can vary, which may result in large phenotypic variability and changes in penetrance.

Although we know very little about Mendelian rare disease modifiers, it is evident that phenotypic variability resulting from modifiers is an important aspect to be considered and researched [16,17,18,19]. For example, as discovered almost one and a half centuries ago (1882), Gaucher disease (GD), one of the rare Mendelian disorders resulting from mutations in the *GBA1* gene, is known to have a wide spectrum of phenotypes even among siblings. However, despite decades of research efforts, there are major gaps in identification and understanding of genetic modifiers in Gaucher disease. Addressing these gaps for GD [8], as well as other rare diseases, is important for diagnostic, prognostic, therapeutic, and overall patient management procedures.

To completely understand this variability in the phenotypic expression of rare disorders, it is essential to look at the individual genome. The cost of Whole-Genome Sequencing (WGS) has become more economical in recent years, making it feasible to get an almost complete view (~98–99%) of an individual’s genome. WGS data allows the exploration and analysis of all the genetic variants that are present in an individual. Different bioinformatics methods have been developed to identify candidate causal variants for the disease. Because of the advances in bioinformatics methods, it is now feasible to identify causal variants for a phenotype more precisely and accurately than ever before [20,21,22], which opens up the possibility to study modifier variants at the individual level.

In this article, we review the existing challenges of genetic modifier identification with respect to rare Mendelian diseases. We summarize some experimental approaches, as well as computational methods which have been used in genetic interaction studies and discuss how this research area can benefit from further improvements.

## 2. Current State of Rare Disease Genetic Modifier Research

**Figure 1:**
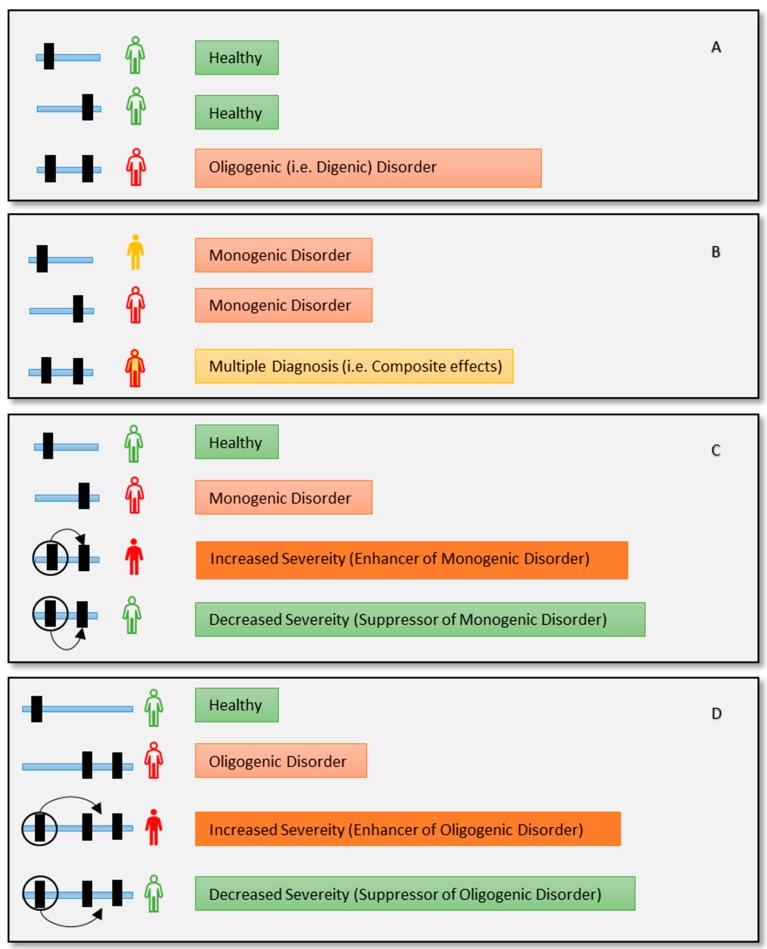
Different genetic phenomena in combination with Mendelian disorders, can make disease characterization challenging. (**A**) This is a case of a digenic disorder. Two individually healthy variants combinedly produce the disease (e.g., Bardet-Biedl syndrome caused by *BBS1* and *BBS10* [35]). (**B**) Two different monogenic disorders may produce a blended or composite representation of both diseases (e.g., Mutation in *NPL* (causing sialic acid disorder) and *GJB2* (causing deafness) creating composite disease phenotype for a patient [36]). (**C**) A case for a genetic modifier. Disease variant is modified by the modifier variant (circled) that enhances or suppresses the severity of the disease (i.e., Spinal Muscular Atrophy modified by *SMN2* variants [37]). (**D**) An oligogenic disease can be modified by a modifier variant as well (i.e., Digenic Usher syndrome modified by *PDZD7* [38]). More complex scenarios are also possible, such as multiple modifier alleles that can act independently or together (joint effect) [31].

The definition of monogenic and oligogenic disorder is based on the number of the primary disease-causing genes. This does not necessarily mean that the relation between genotype and phenotype is straightforward, especially due to different phenomenon such as epistasis, genetic interactions, or modifications [23,24]. All these phenomena can be referred to as the change of phenotypic outcome of one variant by another variant [25]. However, each of these terms can mean a specific phenomenon based on its context. For example, a mono/oligogenic disease can also be modified by other mutations causing incomplete penetrance and variable expressivity [26,27,28] (Figure 1). Genetic modifier can be involved in modifying pleotropic phenotype [29,30,31]. These modifiers may result in a different/novel phenotype. Disease characterization becomes even more challenging for individuals carrying two or more monogenic disorders that may have overlapping (resulting in blended) or different (resulting in composite) phenotypes each of which may be modifiable [7,19] (Figure 1). As we are discussing the effect of genetic modifiers on monogenic disorders, it is essential to clarify why the disease is still referred to as monogenic while multiple variants are involved.

### 2.1. Oligogenicity and Genetic Modifiers

Oligogenicity refers to the multigenic inheritance (involving two or more genes), regardless of modifiers [27,32,33,34]. More specifically, oligogenic diseases are caused by the co-occurrence of mutations in two or more genes. All causal variants must be present in a patient’s genome for oligogenic disease presentation; having a variant in just one gene is not sufficient for the disease manifestation [25] (Figure 1A). In contrast, it may be also possible that one individual has more than one genetic diagnosis resulting in composite or blended phenotype (Figure 1B). On the other hand, in the case of modifiers, pathogenic variant/s are enough to cause the disease (i.e., disease causing) while the other variant/s in modifier genes make the disease outcome more (enhancer) or less (suppressor) severe (Figure 1C,D). Genetic modifiers by themselves may not result in any phenotype and may be rare or common in an untargeted population.

Although some tools and public databases of oligogenic diseases also include genetic modifiers [39,40], a clear distinction between them is necessary for accurate clinical decision making. Recently, researchers have found a genetic modifier in a two-month-old girl who was diagnosed with left ventricular noncompaction (LVNC), a rare heart condition that is caused by heterozygous pathogenic variants in *MYH7* and *MKL2* genes [41]. Both variants were previously unknown. To find out the genetic cause, the authors performed Whole-Exome Sequencing (WES) of the proband and her family and found that the girl is carrying the same disease-causing variant combination as her father (i.e., oligogenic disease). While her father showed no significant sign of the disease at the age of 37 years, his daughter had severe symptoms at just three months of age. Their first child died as a fetus with failure in biventricular noncompaction and was carrying these same three variants. They also found that her 4-year-old sister was also carrying the causal variant combination and is an LVNC patient. To find out the source of this increased severity, the authors performed hierarchical filtering for candidate modifiers and found the existence of a modifier variant (enhancer, Figure 1D) which came from the unaffected mother. The variant was found in gene *NKX2-5* with the allele frequency of 0.0009792 on gnomAD [42]. They confirmed their findings with the aid of a mouse model and induced pluripotent stem (iPS) cells.

This study shows how genetic modifiers can modulate the effect of oligogenic diseases as well (Figure 1D). It is evident that with the integration of appropriate genomic assessment, clinicians may provide more accurate patient management [36]. Furthermore, integrating genetic modifiers in genome assessments holds great promise to further help with patient management, but also to facilitate better diagnosis and prognosis of disease progression [43].

Studies have been done to develop tools to prioritize the oligogenic variants that are responsible for rare diseases. Recently, OligoPVP [39] and VarCoPP [44] were developed in an attempt to prioritize two or more variants that together cause the disease.

OligoPVP was developed based on the improved version of PhenomeNET Variant Predictor (PVP) [45]. PVP scores disease-causing variants from WGS or WES data based on its pathogenicity. The authors used six numeric and categorical features like CADD [46], DANN [47], GWAVA [48], PhenomeNET scores [49], disease inheritance mode, and genotype, along with 54 binary attributes. Together these attributes are used to train a Random Forest (RF) classifier to classify variants as causative or noncausative. Recently, the authors developed an improved version of PVP—DeepPVP [50]. It uses seven additional features. These 76 features are processed through a Deep Artificial Neural Network model to produce a score for each variant which denotes the variant’s predicted pathogenicity. OligoPVP is built on DeepPVP. OligoPVP combines and compares DeepPVP scores of two or more variants to prioritize disease-causing pairs/triplets.

VarCoPP is a more recent tool that also uses an RF classifier to classify variants. Their classifier uses 11 features and is strictly designed to process alleles in pairs. For example, there are four CADD scores among these 11 attributes. Each of these four attributes are strictly designed to process the CADD score of four alleles from a pair of genes. While VarCoPP was able to identify 20 out of 23 di-genic combinations from DIgenic diseases DAtabase (DIDA) [40] fused with 1000 Genomes Project [51] data (a >80% support score), a limitation of the tool is that it works best when the number of candidate variants is less than 150. 

Of note, these two tools were trained with a small number of samples size. Both VarCopp and OligoPVP use DIDA as a source of their training variant set. DIDA is primarily focused on di-genic diseases, where two independent mutations co-occur to produce a disease phenotype (Figure 1A). The DIDA database does contain some modifier entries which might be useful for training a tool to detect genetic modifiers and the authors of OligoPVP proposed that OligoPVP may be able to identify modifier interactions. However, both OligoPVP and VarCoPP techniques and training features are mostly focused on pathogenic effects caused by a combination of multiple variants. Since modifiers do not necessarily have disease-causing properties, they may be left unrecognized by these tools. Therefore, the effectiveness of these tools in modifier discovery, especially for genetic suppressor identification, needs further assessment.

### 2.2. Genetic Modifier Studies in Literature

**Figure 2:**
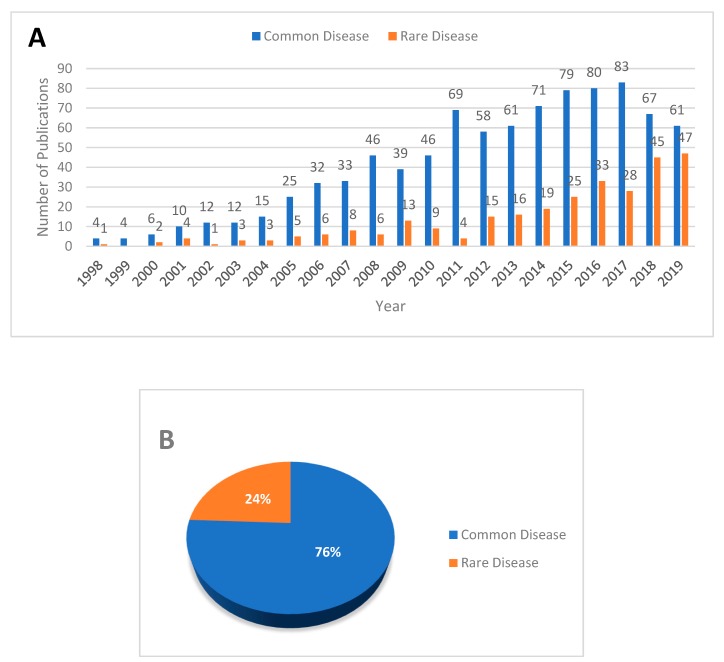
Published literature on modifier studies from 1998 to 2019. Keyword-based literature search result was extracted from PubMed and WoS (See Figure S1). (**A**) Histogram showing a comparative view of published literature for each year. (**B**) Pie chart showing a comparison between common and rare disease studies from 846 literature records.

An assessment of the search result of published literature shows that interest in the genetic modifier study has substantially increased in the last decade (2010–2019: 916 records) than the previous (1998–2008: 281 records) (Figure 2). The data was collected by searching PubMed and Web of Science (WoS). The search logic and document filtration process are described in Figure S1. So far, the effect of genetic modifiers has been predominantly assessed for common diseases via genome-wide association studies (GWAS), whereas the identification of genetic modifiers in rare diseases has not been extensively studied. For instance, our assessment of the extracted literature shows that only 24% of the studies are focused on rare diseases (Figure 2B).

To explore the current knowledge on genetic modifiers of rare diseases, we explored the Online Mendelian Inheritance in Man (OMIM) database [52], which reports the curated information on Mendelian disorders. We extracted only 31 modifier entries for both common and rare disorders from OMIM. To get only rare Mendelian disease entries, we used the rare diseases list from OrphaNet [53]. In the end, we found only 24 rare Mendelian disease modifier entries (Table 1). This finding shows the scarcity of knowledge of Mendelian disease modifiers. While modifier studies are predominantly done on common disorders (Figure 2B), we find that OMIM has more modifier entries for rare diseases (24 entries) compared to common diseases (7 entries). This could be because most common diseases are often non-Mendelian complex disorders, which are not included in OMIM, as well as the nature of GWAS results.

NeuroGeM is one of the earliest databases on genetic modifiers specific to Neurodegenerative disorders [54]. The database was built by combining knowledge gained from *Drosophila melanogaster*, *Caenorhabditis elegans*, and *Saccharomyces cerevisiae* resources. Recently, Sun et al. developed a manually curated database for genetic modifiers named PhenoModifier [55]. The authors sifted through publications to produce the database. They provide a web-based query platform to search modifiers by disease name, gene, or variant.

## 3. GWAS and Genetic Interactions

Genetic modifiers are often found in a functionally similar pathway of the target gene because of possible genetic interactions [41,56,57,58,59,60,61,62]. Hence, genetic interaction analysis plays a crucial role in identifying genetic modifiers. A range of bioinformatics tools have been developed to predict bi-locus SNP interactions from Single Nucleotide Polymorphisms (SNP) array data of large populations [63,64]. These tools are designed to capture “statistical epistasis” for the traits that are common in the population.

Historically, Bateson was the first person who introduced the term ‘epistasis’ in 1909 [65], referring to it as ‘masking’ the ‘effect’ of another independent locus. The characterization of the ‘effect’ of a locus, ‘independence of effect’ was not well defined at that time, which created confusion and debate about epistasis among researchers, especially between geneticists and biometricians who studied quantitative traits. That puzzle further expanded when R.A. Fisher first analytically showed that the Mendelian law of segregation is compatible with the biostatisticians’ segregation laws, but struggled to explain ‘dual epistacy’, or the interaction between pairs of loci [66,67]. In the early 1950s, the mystery of different types of epistasis was explored by studying different epistatic components like additive, non-additive and additive-by-additive, additive-by-dominance, and dominance-by-dominance which was explained by a quantitative model [68,69]. Later, the term “epistasis” was used by multiple disciplines like biology, biostatistics, molecular biology, evolutionary genetics, and they have used their area-specific perspective to explain epistasis [70]. Most of these efforts tried to develop a unified statistical model for epistasis, but statistical interaction often fails to describe the biological mechanism of epistasis [67,70,71,72,73,74,75]. This may be partly because statsistical methods tend to analyze interactions using additive models [31,76].

For the last 12 years, GWAS has been the primary approach to study the common genetic control of various common/complex traits in populations. To identify possible statistical epistasis (SNP interactions) from GWAS, researchers have taken different approaches such as exhaustive, stochastic, heuristic, machine learning, and neural network approaches [63,64,77].

Amongst the epistasis identification tools, Multifactor Dimensionality Reduction (MDR) is perhaps the most prominent, which uses an exhaustive method [78]. Exhaustive methods compute all *n*-locus (*n* is the number of SNPs) interactions in a brute-force fashion and search for significant interaction within the permutation space. MDR uses model-free statistical methods and is reported as capable of identifying *k*-way interactions (*k* denotes the level of interactions). GMDR-GPU [79], Model-based MDR (MB-MDR) [80] are a couple of the latest improvements over MDR. BOOST is another popular exhaustive epistatic interaction prediction tool [81], and one of the fastest among the exhaustive methods. Stochastic methods like Bayesian Epistatic Association Mapping (BEAM) determine interactions by randomly sampling the search space [82], and heuristic methods like AntEpiSeeker model the search for epistatic interactions as an ant colony optimization (ACO) procedure where each locus is represented by a certain level of pheromones [83]. All of these tools are designed to take genotype array data as input. Overall, they can handle 100,000 to ~500,000 SNPs from a few hundred to thousands of samples (balanced case-control) within a timeframe of 24 hrs to 60hrs [71,78,80,81,84]. These tools were used in a few independent studies to identify interactions between SNPs that may be associated with a particular phenotypic condition [85,86,87,88,89,90,91,92].

Over the years, researchers have been able to associate thousands of variants with certain diseases/traits through GWAS. However, pinpointing variants using the available GWAS tools is an existing challenge [93]. This shortfall becomes more obvious with challenging attempts of applying GWAS to identify SNP interactions [94,95]. As a result, to date, there is lack of reported experimental validation of the results predicted by these tools in the literature. Additionally, having interaction does not necessarily mean that it would have a modified effect on the phenotype [96]. Functional interaction between two genes also depends on the underlying pathways and their reciprocal dependence [97,98].

Furthermore, these tools are prone to the same technical shortcomings as a typical GWAS when it comes to the discovery of causal rare Mendelian disease variants. Since disease-causing variants are sporadic, and there are not enough data-samples, GWAS fails to capture the true significant alleles for rare Mendelian diseases [99]. Identification of genetic modifiers of a rare Mendelian disease thus has the same consequences because the sample size can be restricted to one or a handful of patients only [100]. Few studies tried to overcome this issue by aggregating patient data to perform GWAS [12,101,102]. Nevertheless, even if we were to collect and combine rare disease patients’ genotypes, we must ensure the privacy of genome information [99,103,104,105,106].

## 4. WGS Approach for Modifier Studies

WGS analysis offers a more comprehensive view of the genome than genotyping array by giving access to almost all of the genetic variants that are present in an individual (coding and non-coding regions). Thus, the exploration of the individual genetic variation is not restricted to single nucleotide variants (SNVs), as it allows the identification of structural variants (SVs) as well [2,19,107,108]. Moreover, continuous improvements in knowledge of the transcriptome, known interactions within and between different biological entities, related ontologies, and bioinformatics tools are providing a better understanding and more precise annotation of the variant effects.

**Figure 3:**
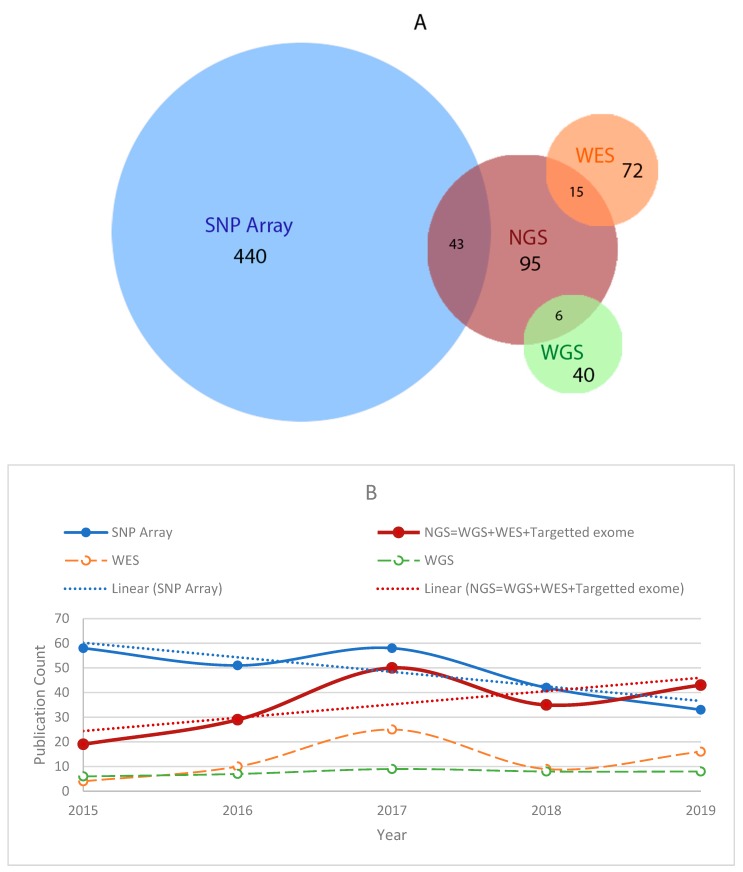
A comparative view of the usage of a SNP array and sequencing technology to study genetic modifiers. (**A**) Publication count between the years 1998–2019. (**B**) Recent (2015–2019) publications indicate increased interest in high throughput sequencing techniques. Two linear (dotted) lines are used to show the trend shift.

**Figure 4:**
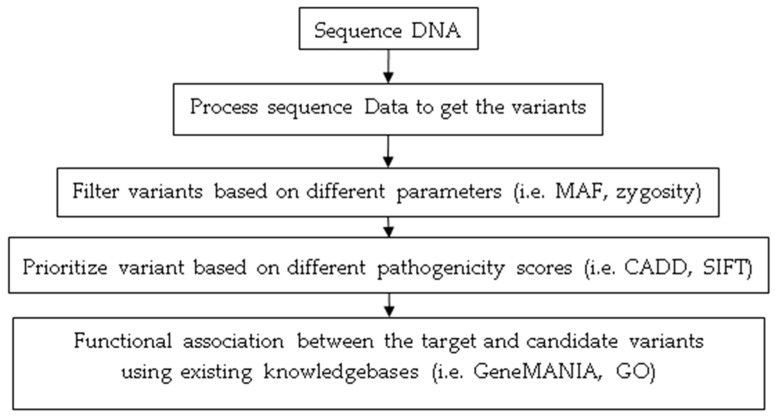
Typical analysis steps followed to identify modifiers using NGS based approaches in recent years.

Because of these advances, it is now feasible to identify causal variants for a phenotype more precisely and accurately, opening up the prospect of studying genetic modifier variants at the individual level. As a result, WGS analysis has become a promising approach to study rare diseases genomics and modifiers, where individual genome analysis is essential [2,4,109,110]. Our literature analysis also indicates how rare disease modifier studies have risen significantly in the last five years as WGS becomes increasingly more affordable (Figure 3B). Among the total 418 studies from 2015–2019, 42% of studies used next-generation sequencing (NGS) data analysis, which includes WGS, WES, and targeted exome sequencing. To get an insight into the recent approaches of modifier studies for Mendelian disorders, we took a more in-depth look into the NGS based studies. Most of these NGS based approaches performed parameterized variant filtering, pathogenicity score-based prioritization (based on scores from pathogenicity scoring tools), and lastly, functional association to identify the modifier variant(s) (based on functionally annotated ontologies and knowledgebases) (Figure 4). These approaches often rely on manual variant prioritization from candidate variants by human. In such process, human experts infer functional similarity between the candidate variants and the target causal mutation by reviewing relevant knowledge-bases [41,111,112,113]. Incorporation of a computational method that can comprehensively analyze and prioritize modifiers from candidate modifier variants is still an open area for research.

Another advantage of using WGS in a modifier study is that it allows exploration of almost all the variants in a genome and also allows comprehensive analysis. This is particularly helpful when the same gene can modulate the phenotype differently depending on the type of variant. For instance, spinal muscular atrophy (SMA) is caused by the *SMN1* gene. A similar gene *SMN2*’s copy number acts as a dosage suppressor of *SMN1*. However, Prior et al. reports that it is possible to suppress SMA by having homozygous c.859G>C transversion mutation in exon 7 in the *SMN2* gene [37]. The mutation replaces glycine by arginine at codon 287. This change produces an exonic splicing enhancer element and increases the amount of full-length *SMN2* transcripts which is similar to the *SMN2* copy-number that produces a less severe SMA phenotype.

## 5. Experimental Approaches to Detect Genetic Modifiers

Multiple reviews discuss the role and importance of model organisms in rare disease studies [25,114,115,116]. Most of the experimental procedures for identifying rare disease modifiers use model organisms because of the advantage of experimental reproducibility through inbred strains that have the same genetic background allowing comparative assessment to identify modifiers [117].

Among all model organisms, the single-celled Yeast has one of the most comprehensive interaction networks produced by a high-throughput reverse genetic approach [118]. On the other hand, organisms like *C. elegans* have the advantage of being a tractable multi-cellular organism with biological simplicity and elegant genomics. *C. elegans* shares extensive genome conservation with humans, which supports the translatability of the findings [119]. One particular limitation for any model organism is that not all human genes have orthologs in any given organism. For instance, 35% of human genes do not have any specific orthologs in flies [120]; however, this number decreases when moving to vertebrate models such as zebrafish or mice. In fact, zebrafish and mice offer a more robust phenotypic comparison [114,121,122]. However, a simpler organism (i.e., yeast, *C. elegans*) allows high-throughput screening for interaction and modifier discoveries, as reflected by the number of interactions discovered in these model organisms and compiled in BioGrid [118].

### 5.1. Reverse Genetic Screens

One of the most comprehensive knowledgebases of genetic interaction is available for yeast (*S. cerevisiae*). According to BioGrid statistics (accessed on October 7, 2019), there are 441,949 unique genetic interactions for yeast alone, which is 65% of the total known genetic interactions for all organisms combined (684,244). One of the major contributions to this yeast interaction knowledge base comes from a reverse genetic approach using synthetic gene array (SGA) screens [123] that looked for synthetic lethal interaction initially [14,29], but also recently found suppressors [117,124].

There are other experimental methods like Epistatic Mini-Array Profile (eMAP) [125] and RNA interference (RNAi) [126] to test for interactions [77,127]. In eMAP, a subset of genes is chosen (e.g., genes belonging to a given pathway or process), analyzed pairwise in an array, and then clustered to find out synthetic lethal interactions [125,128]. A more widely used approach to analyze interactions is RNAi, where instead of completely knocking out the gene function, RNAi libraries are used to knock-down the expression of a gene of interest. Multiple studies have performed RNAi on *C. elegans* strains to test for interactions [129,130]. However, their efficiency in terms of scale was lower than the yeast’s SGA experiments [77]. Noise in large-scale RNAi is one of the reasons for this lower efficiency; off-target effects and variable knockdown efficiencies are common RNAi complications [127,131,132].

### 5.2. Forward Genetic Screens

In reverse genetic approaches, researchers modify a known gene of interest and observe the phenotypic change (unknown phenotype). In contrast, the forward genetic approach tries to find out the genetic cause (unknown gene/variant) for a particular phenotype (known phenotype). With forward mutagenesis, it is possible to study the genetic modifier unbiasedly, allowing the exploration of modifiers beyond the known variants [122]. Chemical mutagens like Ethyl methanesulfonate (EMS) and N-ethyl-N-nitrosourea (ENU) are two widely used mutagens for the forward genetic screen. There is no strict rule regarding the use of each mutagen, but genetic studies in plants, fruit flies and *C. elegans* preferentially use EMS, whereas ENU is used in mice and zebrafish [60,114,133,134,135,136,137]. Both EMS and ENU can induce random point mutation; EMS produces G/C base pair mutations and ENU has a bias toward A/T base pairs. In high-throughput mutagenesis screens, a cocktail of EMS and ENU is also used [138].

## 6. Prospects and Challenges of Computational Model in Modifier Identification

Continual increases in computational power is allowing researchers to run powerful machine learning algorithms and advanced deep learning models [139,140]. Overall improvement of the core algorithms for regression, classification, and clustering analysis has significantly improved applied bioinformatics for medical science, health science, and genetics [141,142]. Even with these advancements, the resources for detection and prioritization of modifier variants remain scarce. A computational model that can identify genetic modifiers from the individual genome by aggregating known information can become a valuable resource for the diagnosis and treatment of patients.

A review on Hereditary spastic paraplegias (HSPs) [143] and another very recently on Charcot-Marie-Tooth (CMT) [27] discuss how genetic modifier studies are uncovering the ‘missing heritability’ for these rare Mendelian disorders. The authors argued that despite being a monogenic disorder, non-Mendelian factors are essential to consider to overcome the diagnostic gap and to provide better therapeutics. Genetic modifiers work through a complex interaction system, most of which are yet to be discovered [56]. In another review, Deltas C. proposed the hypothesis of the ‘Alpha effect’ of genetic modifiers in disease diagnosis and patient management [144]. According to the hypothesis, with a proper combination of diverse knowledgebases, we could potentially measure the severity or contributory role of a particular variant in an individual to a specific phenotype or disease. While identifying the negative effect of the modifiers (enhancers) will help to provide better diagnostics insight, identifying positive modifiers (suppressors) beyond diagnostic may also help researchers to develop personalized therapies.

Over the years, researchers have developed such knowledge bases and ontologies, which have facilitated the development of computational models and automation systems for bioinformatics analysis. A range of scoring and variant interpretation techniques have been developed to better understand each variant and its pathway [145]. Resources like BioGrid [118], GeneMania [146], model organism-specific knowledgebases [29,147], Gene Ontology [148,149], Phenotype Ontology [150], and pathway analysis [151] are currently helping researchers in modifier identification studies and may help to develop computational models for modifier identification in the future [2]. With all these resources available, a possible challenge to capture or measure the alpha effect or modifier effect would be identifying the appropriate feature combinations.

## 7. Discussion

The identification of genetic modifiers is an existing challenge, especially for rare diseases. It can be seen as a two-front challenge—experimental and computational. The experimental challenge includes establishing high-throughput forward genetic screening techniques utilizing model organisms as current mutagenesis and screening techniques often take years to identify modifiers [152,153,154]. On the other hand, the computational challenge exists in identifying genetic modifiers by comprehensively analyzing all the variants from an individual genome [155,156].

Phenotypic heterogeneity and variability is a major concern for rare Mendelian disorders, which often leads to misdiagnosis and/or delayed diagnosis [19,114,157,158]. However, naturally occurring suppressor modifiers that reduce the severity or rescue an individual from the adverse effect of a disease-causing mutation can guide researchers and clinicians to its potential therapeutics [8,117]. Phenotypic variability resulting from suppressor modifiers is less studied but presumably is a common phenomenon [117]. Therefore, future research efforts in the area of high-throughput modifier identification, in combination with experimental and computational approaches will not only help with the better diagnosis of Mendelian diseases in a time-efficient manner, but it may also provide direction to its potential therapeutics, which we are significantly lacking [159].

## Figures and Tables

**Table 1:** Modifier gene for the rare Mendelian disorder found in the Online Mendelian Inheritance in Man (OMIM) database.

OMIM	Modifier Gene	Disease	PhenoModifier Gene ID
107670	*APOA2*	Hypercholesterolemia, familial	-
108733	*ATP2B2*	Deafness, autosomal recessive 12	491
112261	*BMP2*	HFE hemochromatosis	-
120353	*MMP1*	Epidermolysis bullosa dystrophica	4312
132811	*EPHX2*	Hypercholesterolemia due to LDLR defect	-
147570	*IFNG*	TSC2 angiomyolipomas	3458
155555	*MC1R*	Albinism, oculocutaneous, type II	4157
168461	*CCND1*	von Hippel-Lindau syndrome	-
190180	*TGFB1*	Cystic fibrosis lung disease	7040
600451	*AKR1C4*	46XY sex reversal 8	-
600837	*GDNF*	Pheochromocytoma	2668
600946	*GHR*	Hypercholesterolemia	-
601627	*SMN2*	Spinal muscular atrophy	6607
602421	*CFTR*	Bronchiectasis with or without elevated sweat chloride 1	1080
603415	*SCN9A*	Dravet syndrome	6335
605204	*TOR1A*	Dystonia-1, torsion	1861
608124	*XYLT1*	Pseudoxanthoma elasticum	64131
608125	*XYLT2*	Pseudoxanthoma elasticum	64132
608845	*ARL6*	Bardet-Biedl syndrome 1	-
609884	*TMEM67*	Bardet-Biedl syndrome 14	-
610162	*CCDC28B*	Bardet-Biedl syndrome 1	79140
610230	*TRMU*	Deafness, mitochondrial	55687
611089	*MTMR14*	Centronuclear myopathy	-
612971	*PDZD7*	Retinal disease in Usher syndrome type	79955

## Supplementary Materials

The following are available online at https://www.mdpi.com/2073-4425/11/3/239/s1, Figure S1: Schematic diagram of the literature review and the textual analysis process. Textual analysis is performed using NVivo 12 plus.
