# Supplementary material for: Genetic Modifiers and Rare Mendelian Disease
Source: Genes (Basel). 2020 Feb 25;11(3):239. doi: 10.3390/genes11030239 (PMC7140819; doi:10.3390/genes11030239)
Supplement: Supplementary file 1 [file genes-11-00239-s001.pdf]

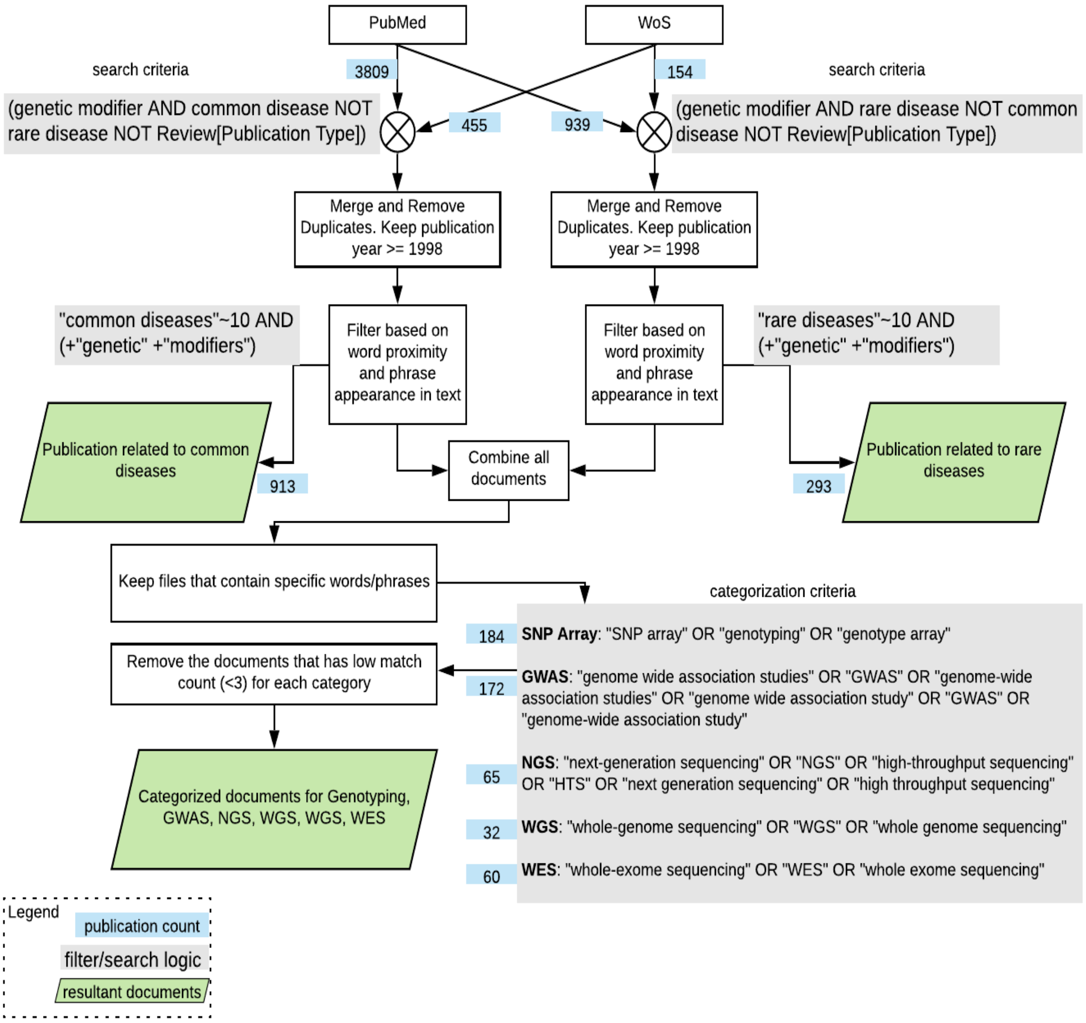

Figure S1. Schematic diagram of the literature review and the textual analysis process. Textual analysis is performed using NVivo 12 plus.
